# Histone acetylation modulators in breast cancer

**DOI:** 10.1186/s13058-025-02006-9

**Published:** 2025-03-31

**Authors:** Xueying Yuan, Jeffrey M. Rosen

**Affiliations:** Department of Molecular and Cellular Biology, Baylor College of Medicine, 1 Baylor Plaza, Houston, TX USA

**Keywords:** Epigenetics, Histone acetylation, Myeloid cells

## Abstract

Breast cancer is the most prevalent cancer in women worldwide. Aberrant epigenetic reprogramming such as dysregulation of histone acetylation has been associated with the development of breast cancer. Histone acetylation modulators have been targeted as potential treatments for breast cancer. This review comprehensively discusses the roles of these modulators and the effects of their inhibitors on breast cancer. In addition, epigenetic reprogramming not only affects breast cancer cells but also the immunosuppressive myeloid cells, which can facilitate breast cancer progression. Therefore, the review also highlights the roles of these immunosuppressive myeloid cells and summarizes how histone acetylation modulators affect their functions and phenotypes. This review provides insights into histone acetylation modulators as potential therapeutic targets for breast cancer.

## Background

Breast cancer is the most common cancer among women globally [1]. Breast cancer can be classified by the expression of estrogen receptor (ER) and progesterone receptor (PR) and amplification of human epidermal growth factor receptor 2 (HER2) [2]. Breast cancer that lacks those biomarkers is categorized as triple-negative breast cancer (TNBC) [3, 4].

Epigenetic changes contribute to tumorigenesis, progression, and metastasis of breast cancer [5]. They can also affect the tumor-associated immune cells, which play important roles in tumor growth and treatment response [5, 6]. Therefore, many therapeutics have been developed to target epigenetic factors in breast cancer [7]. Histone acetylation is one of the most important epigenetic modifications. Histones are critical components of nucleosomes. Each nucleosome contains two subunits made of H3, H4, H2A, and H2B histones. Each histone contains a tail enriched with lysine (K) residues, which can be acetylation sites. Acetylation of histone tails can increase chromatin accessibility at the enhancer, promoter, and transcribed regions and thus promote gene transcription [8] (Table 1). The “writers” of histone acetylation are histone acetyltransferases (HATs) categorized into four major families Gcn5-related N-acetyltransferases (GNATs), MYST, CREB-binding protein (CBP)/E1A-associated protein p300 (EP300) and steroid receptor coactivators (SRCs) [9, 10]. Histone acetylation is removed by histone deacetylases (HDACs), which include classical HDACs and sirtuins with different cellular localization (Table 2) [11]. Histone acetylation also acts as a signal recognized by “readers” bromodomains (BRDs), and many chromatin-modulating proteins including HATs can contain BRDs [12]. In addition, both HDACs and HATs can have non-histone targets such as transcription factors (Tables 1 and 2).

Inhibitors of both writers and erasers of histone acetylation have been investigated as potential therapeutics for breast cancer [13]. However, very few published reviews have an in-depth focus on these modulators. Therefore, here we provide a comprehensive review of current findings on histone acetylation modulators and their inhibitors in breast cancer. In addition, we also summarize the roles of tumor-promoting myeloid cells in breast cancer and discuss how they can also be regulated by the histone acetylation modulators.

## HATs in breast cancer

HATs have been reported as both oncogenes and tumor suppressors in many cancer types including breast cancer [14, 15]. Histone H4K8 acetylation by KAT2B, a GNAT family HAT, reduced replication fork stability in breast cancer cells in vitro, and reduced levels of KAT2B may predict PARP inhibitor resistance [16]. KAT2B also inhibits proliferation of p53 mutant breast cancer cells in vitro by acetylating p53 and histones [17]. KAT5, a HAT of the MYST family, has been identified as a haploinsufficient tumor suppressor, and loss or low expression of KAT5 was observed in a fraction of breast cancer cases, correlating with poor prognosis [18, 19]. Another study showed that low expression of KAT5 led to decreased H3K4 acetylation and knockdown of KAT5 promoted the progression of MDA-MB-231 xenografts, a TNBC model, but not MCF-7 xenografts, an ER-positive breast cancer model [20]. This indicates that the role of KAT5 in breast cancer is complex and context dependent.

Compared to the tumor-suppressing role, more evidence has been found regarding the tumor-promoting roles of various HATs. Acetyltransferase activity of KAT7 (MYST family) was found to facilitate radiotherapy resistance in breast cancer cells in vitro through activation of the PI3K/AKT pathway [21]. KAT2B, EP300, KAT6A (MYST family), and KAT2A (GNAT family) are recruited to ER-responsive promoters and are critical for estrogen-dependent proliferation of ER-positive breast cancer cells [22–24]. KAT6A was found to be frequently amplified and/or overexpressed in breast cancer and has been correlated with worse prognosis in ER-positive breast cancer patients [24, 25]. Moreover, the silencing of ATF2 (GNAT family) reduced the expression of genes associated with endocrine therapy resistance in ER-positive breast cancer cells in vitro [26]. The SRC family HATs are transcription coactivators for steroid hormone receptors including ER and PR and can acetylate steroid hormone receptor-responsive promoters [27–30]. SRC-1 and SRC-3 also facilitate endocrine therapy resistance and activate breast cancer-promoting genes in an ER-independent manner [27, 31]. In addition, CBP/EP300 also activates transcription of the androgen receptor (AR) and thus promotes AR signaling in AR-positive breast cancer model MDA-MB-453 in vitro and in vivo [32].

Besides activating the transcription of hormone receptor-responsive genes, HATs can also promote epithelial-mesenchymal transition (EMT). EMT describes a process in which epithelial cells lose their polarity and junctions to gain mesenchymal traits [33]. It is associated with breast cancer invasion, migration, metastasis, and stem-cell-like phenotypes [34]. In TNBC, enrichment of the EMT gene signature was found in residual tumors after neoadjuvant chemotherapy [35]. Multiple HATs were found to be involved in EMT activation. KAT2A induced EMT in breast cancer cells by activating the transforming growth factor-β (TGF-β)/Smad pathway, and inhibition of KAT2A reduced the survival, migration, and invasion of MDA-MB-231 cells in vitro [36]. KAT5 was shown to acetylate the key EMT-inducing transcription factor (EMT-TF) Twist to promote transcription of EMT genes in vitro in basal-like breast cancer cells HEK293 and SUM1315 [37]. In addition, EP300 induces the expression of key EMT regulators in non-tumorigenic breast epithelial cells MCF10A by histone H3 acetylation and interacting with other transcription factors such as c-Myc [38].

**Fig. 1:**
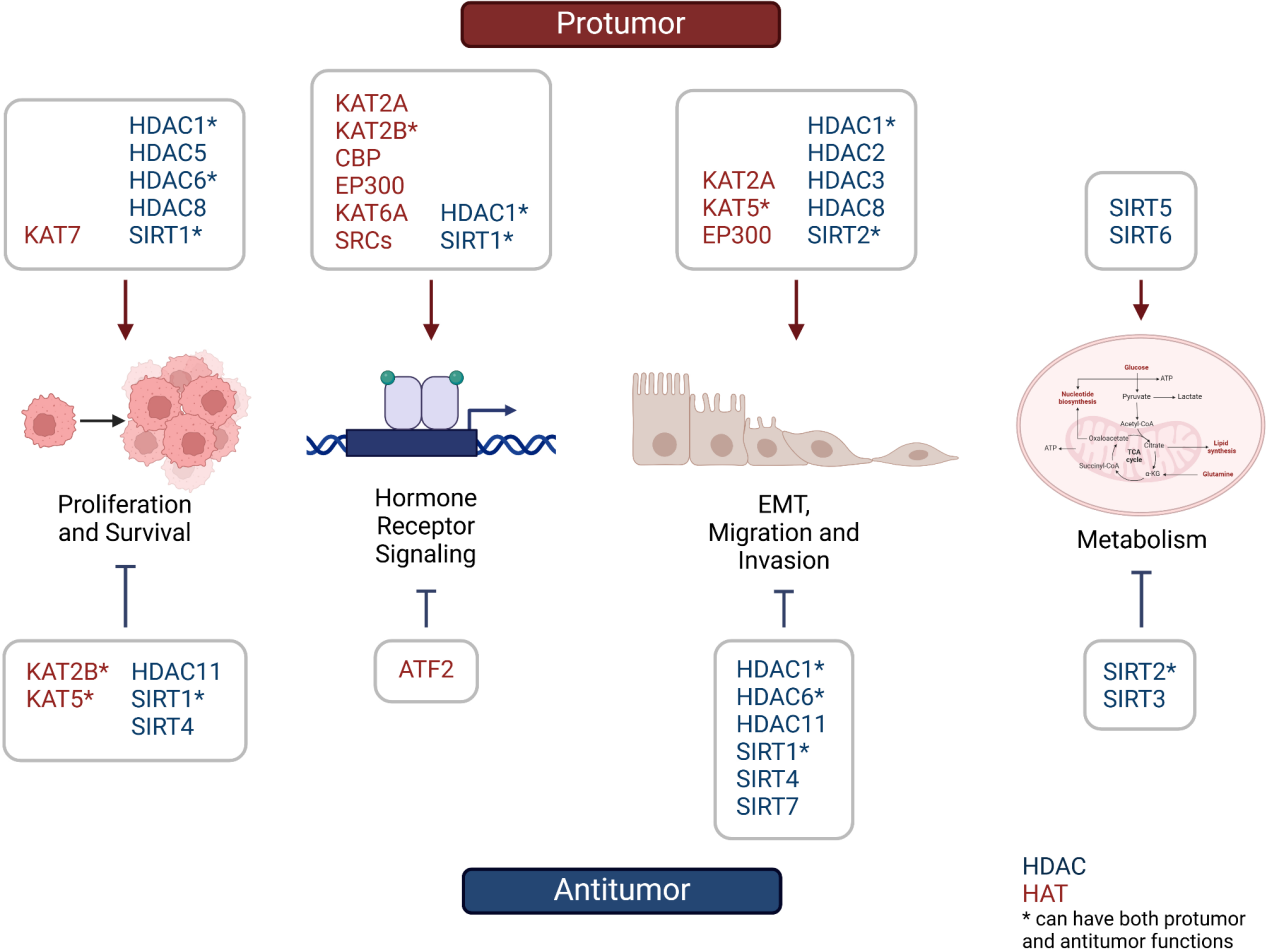
Protumor and antitumor functions of HATs and HDACs in breast cancer

In summary, the functions of HATs have been primarily studied in hormone receptor-positive breast cancer, as HATs were known to regulate the transcription of hormone receptor-dependent genes (Fig. 1). In contrast, studies investigating other roles of HATs were mostly conducted in breast cancer cell lines in vitro. More in vivo studies will be needed to further elucidate the roles of HATs in breast cancer, especially the hormone receptor-independent subtypes.

**Table 1:** The targets and functions of histone acetyltransferases (HATs)

Targets	HAT family	Functions
Histones: H2A, H2B, H3, H4	All families	• Increase chromatin accessibility • Promote transcription activation [29, 39, 40]
Transcription factors (e.g. p53, STAT3, c-Myc, cyclins, PTEN)	GNATs (KAT2A, KAT2B) CBP/EP300 MYST (KAT5)	• Increase or decrease the affinity of transcription factors to DNA (site-dependent) [40, 41] • Promote interaction and transactivation [40, 42] • Prevent or promote ubiquitination and degradation [40]
Nuclear receptors	CBP/EP300 MYST (KAT5)	• Facilitate transactivation of the receptors [43, 44] • Regulate ligand sensitivity [45]
DNA repair proteins (Ku70, ATM)	CBP GNATs (KAT2B) MYST (KAT5)	• Activate ATM activity in response to DNA damage [46] • Disrupt Ku70 interactions to promote apoptosis [47]

### HAT inhibitors in breast cancer

HAT inhibitors have been developed and shown to have antitumor efficacy in many cancer types including breast cancer. Most current studies focus on inhibitors of CBP/EP300 and KAT6A/KAT6B, MYST family HATs [48].

CBP/EP300 HAT inhibitors CPI-1612 and A-485 were shown to inhibit the growth of ER-positive breast cancer in vitro and in vivo by reducing ER-dependent gene expression [49, 50]. However, inhibitors of the CBP/EP300 HAT domain were not selective and not successful in the clinic. In contrast, inhibitors of their BRD have recently shown promising results [51]. BRD inhibition can reduce acetylation by the HAT domain, but compared to HAT inhibition, BRD inhibition led to an attenuated effect and decreased acetylation at some unique sites [52]. In ER-positive breast cancer, the CBP/EP300 BRD inhibitor GNE-049 had similar effects as A-485 in downregulating the expression of ER-dependent genes and inhibiting cancer cell proliferation [50]. In TNBC cells, another CBP/EP300 BRD inhibitor I-CBP112 reduced drug efflux by repressing ATP-binding cassette transporters and sensitized the cells to chemotherapies in vitro [53]. The CBP/EP300 BRD inhibitor FT-6876 reduced AR signaling and inhibited the growth of AR-dependent breast cancer models in vitro and in vivo [32]. Our recent publication demonstrated that CBP/EP300 BRD inhibitor IACS-70,654 reduced the proliferation and inhibited the metastasis of neutrophil-enriched TNBC in vivo [54]. However, currently Inobrodib is the only CBP/EP300 BRD inhibitor in early clinical trials for treating solid tumors, including breast cancer [48]. It showed promising results in early clinical trials for hematological malignancies and prostate cancer, but its effects on breast cancer models/patients have not been published [55, 56].

In addition to CBP/EP300 inhibitors, the KAT6A/KAT6B inhibitor CTx-648 demonstrated antitumor activity in vivo in ER-positive breast cancer models with resistance to endocrine therapy [57]. PF-07248144 is the KAT6A/KAT6B inhibitor currently in clinical trials, and the results from the phase 1 clinical trial were recently published and showed durable antitumor effects in metastatic ER-positive HER2-negative breast cancer [58].

Taken together, HAT inhibitors have been tested in breast cancer, and two inhibitors have entered early phase clinical trials. Most studies of HAT inhibitors focused on hormone receptor-positive breast cancer, most likely because the roles of HATs in hormone receptor signaling have been more extensively studied. HAT inhibitors may also be suitable for treating hormone receptor-independent breast cancer, but more preclinical studies might be needed before more inhibitors can enter clinical trials.

### HDACs in breast cancer

HDACs have been targeted for the treatment of many cancer types including breast cancer because of their role in many biological functions associated with tumor progression [59–61]. In the clinic, metaplastic breast cancer, an aggressive and treatment-resistant subtype, was found to have elevated HDAC activity [62]. Among all HDACS, Class I HDACs were most extensively studied. High expression of HDAC1 has been associated with high ER and PR expression in multiple studies [63–66]. In contrast, HDAC2 expression was found to be significantly higher in hormone receptor-negative breast tumors [64]. In ER-negative breast cancer, studies suggested that HDAC1 can suppress the expression of ER and its associated genes to promote their growth, indicating its complex functions [67, 68]. HDAC1 was also shown to induce proliferation and migration of breast cancer cells by upregulating Interleukin (IL)-8 signaling [69]. Induced cytoplasmic expression of HDAC3 has been associated with brain metastasis in breast cancer patients [70]. HDAC1, HDAC2, and HDAC8 were found to form a complex with EMT-TF Snail and induce EMT in breast cancer cells to promote migration [71–73]. However, HDAC1 was also demonstrated to downregulate Wnt signaling to reduce migration and invasion in breast cancer cells [74]. In addition, HDAC2 and HDAC3 were shown to facilitate the inhibition of vascular endothelial growth factor (VEGF) signaling, which promotes angiogenesis to support tumor progression, in breast cancer cells in vitro [75]. These seemingly contradictory results can be explained by the study illustrating that HDAC1 has distinct substrates in different breast cancer cell lines, highlighting the effects of tumor heterogeneity on HDAC functions [76].

Compared to Class I HDACs, the functions of Class II and IV classic HDACs are not as well characterized, but have also been studied in the breast cancer setting [11]. The loss of HDAC5 induced the expression of cell-cycle genes and thus led to cyclin-dependent kinase (CDK) 4/6 inhibitor resistance in breast cancer [77]. In addition, HDAC5 was shown to deacetylate SOX9 to promote c-Myc expression and help drive endocrine therapy resistance in ER-positive breast cancer in vitro [78]. HDAC6 expression has been correlated with reduced cell motility and better response to endocrine therapy in ER-positive breast cancer cells in vitro [79, 80]. In inflammatory breast cancer, however, HDAC6 was found to have aberrantly high activity and was essential for cell viability [81]. A recent study found induced HDAC6 activity in about 30% of breast cancer patients analyzed and suggested that HDAC6 deacetylates c-Myc to reduce its degradation, contributing to tumor cell viability [82]. Another recent study by Lu et al. demonstrated that phosphorylated HDAC6 induces aberrant chromatin architecture, which supports the tumor growth of TNBC [83]. HDAC11 expression, in contrast, was correlated with better overall survival of breast cancer patients, and HDAC11 knockdown led to enhanced proliferation, migration, and invasion of breast cancer cells in vitro [84]. Nevertheless, another study showed using mouse models that HDAC11 facilitates the growth of breast cancer lymph node metastases while inhibiting the migration from lymph node to distant organs [85].

Besides classic HDACs, sirtuins (SIRT) have also been extensively studied in breast cancer. SIRT1 is overexpressed in ER-positive breast cancer and was shown to promote tumor progression by facilitating ER and estrogen-related receptor signaling [86–88]. SIRT1 was also demonstrated to promote breast cancer formation by interacting with and promoting the activity of AKT [89]. In TNBC, however, SIRT1 is downregulated, and the loss of SIRT1 may promote tumor invasion and survival by impairing lysosomal integrity [90]. Elevated expression of SIRT1 has also been associated with higher rates of metastasis in TNBC but lower rates in all other types of breast cancer [91]. High protein expression of SIRT2 was correlated with poor prognosis in high-grade breast cancer, but the correlation was reversed in intermediate-grade breast cancer [92]. In basal-like breast cancer, SIRT2 can be overexpressed and stabilize EMT-TF Slug to promote tumor invasion and stem-like phenotypes [93]. However, SIRT2 expression was shown to sensitize breast cancer cells to oxidant stress-inducing agents by modulating peroxidase activity [94]. It also was demonstrated to inhibit tumor growth by deacetylating M2 isoform of pyruvate kinase, thus altering glucose metabolism [95]. SIRT3 was correlated with poor prognosis in breast cancer patients, but decreased mitochondrial expression of SIRT3 was associated with poor prognosis [96, 97]. Multiple studies have demonstrated the tumor-suppressing role of SIRT3 in reprogramming cancer cell metabolism in the mitochondria [98, 99]. Distinct from other SIRTs, multiple studies of SIRT4 agreed that it is tumor-suppressive. Decreased SIRT4 has been associated with poor prognosis and induced stemness in breast cancer [100, 101]. In addition, SIRT4 can inhibit IL-6/STAT3 signaling to improve the response of ER-positive breast cancer to endocrine therapy [102]. SIRT5 is another SIRT that plays an important role in cancer metabolism to promote breast cancer progression. SIRT5 can induce the expression of glutaminase and promote aerobic glycolysis in breast cancer [103, 104]. SIRT6 facilitates mammary tumorigenesis by increasing oxidative phosphorylation and has been associated with poor prognosis in HER2-positive breast cancer [105, 106]. SIRT7 can inhibit metastasis of breast cancer by inhibiting TGF-β signaling, and HDAC8 can suppress the expression of SIRT7 to promote cancer cell survival and migration [107, 108].

In summary, HDACs are much more extensively studied in breast cancer than HATs, but most HDACs were found to both inhibit and promote breast cancer depending on the cell context, and some studies reported seemingly contradictory results (Fig. 1). These findings indicate that the functions and targets of HDACs are not the same across all breast cancers and can be dependent on the subcellular location of the HDAC, breast cancer subtype, metastatic status, hormone receptor expression, and tumor grade. Therefore, all those factors will need to be considered when targeting HDACs in breast cancer.

**Table 2:** Family, class, localization, and functions of histone deacetylases (HDACs)

Family	Class	Members	Localization	Function
Classic	Class I	HDAC1, HDAC2, HDAC3, HDAC8	Nucleus only	Deacetylate histones to directly modulate genome accessibility [109]. Deacetylate key transcription factors (e.g. p53) [110, 111].
	Class IIA	HDAC4, HDAC5, HDAC7, HDAC9	Cytoplasm and Nucleus	Scaffold for transcription repression [112]. Deacetylate transcription factor MEF2 [113].
	Class IIB	HDAC6, HDAC10	Cytoplasm (mostly)	Deacetylate and stabilize microtubules [114]. Promote autophagy to mediate cell survival [115].
Sirtuins (SIRT)	Class III	SIRT 1–7	Cytoplasm and Nucleus	Deacetylate histones [116, 117]. Deacetylate transcription factors (e.g. p53, NF-κB) [118]. Deacetylate tubulins [119]. ADP-ribosylation of PARP1 to promote DNA repair [120].
Classic	Class IV	HDAC11	Cytoplasm and Nucleus	Scaffold for transcription repression at *Il10* promoter [121]. Defatty-acylation [122].

### HDAC inhibitors in breast cancer

Although the roles of HDACs in breast cancer are complicated and heterogeneous, many HDAC inhibitors have exhibited antitumor effects in preclinical models of breast cancer [123]. However, to date, no HDAC inhibitor has been approved for the treatment of breast cancer. HDAC inhibitors currently in active clinical trials are vorinostat, belinostat, romidepsin, entinostat, and tucidinostat (Table 3). Vorinostat is a pan HDAC inhibitor and the first HDAC inhibitor approved by the Food and Drug Administration (FDA) [124]. In preclinical models of breast cancer, it was shown to induce apoptosis and autophagy while inhibiting proliferation, EMT, and migration [125]. In addition, vorinostat was found to induce ER degradation and improve the response of ER-positive breast cancer cells to endocrine therapy [126]. Breast cancer patients treated with vorinostat as a single agent failed to show an adequate response in the clinical trial [127]. The published clinical study of vorinostat in combination with endocrine therapy or chemotherapy showed encouraging results, but it never entered late-phase clinical trials for breast cancer (Table 3) [128–130]. Similar to vorinostat, belinostat is also an FDA-approved pan HDAC inhibitor still in early-phase clinical trials for breast cancer [131] (Table 3). In TNBC cells in vitro, belinostat induced cell apoptosis and showed possible synergy with chemotherapy [132]. Belinostat also exhibited synergistic effects with the PARP inhibitor olaparib in *BRCA1*-mutated TNBC cells and xenografts [133]. Currently, no clinical trial results have been published for belinostat.

Romidepsin and entinostat are Class I HDAC inhibitors, different from vorinostat and belinostat. Romidepsin inhibits HDAC1 and HDAC2 specifically and is FDA-approved [134]. In a preclinical model of inflammatory breast cancer, romidepsin treatment led to the destruction of tumor emboli and lymphatic vascular structure, inhibiting the growth of primary tumors and metastases in combination with paclitaxel [135]. In TNBC preclinical models, romidepsin in combination with gemcitabine and cisplatin inhibited tumor growth, EMT, invasion, and metastasis [136]. In contrast, entinostat inhibits HDAC1 and HDAC3 but not HDAC2. The effects of entinostat have been studied across all subtypes of breast cancer. Entinostat was shown to induce the expression of ER in ER-negative breast cancer and sensitize it to endocrine therapy in vitro and in vivo [137]. Entinostat also inhibited tumor-initiating cells in TNBC [138]. In preclinical models of HER2-positive breast cancer, entinostat exhibited combinational synergistic effects with the HER2/epidermal growth factor receptor (EGFR) dual tyrosine kinase inhibitor to inhibit tumor progression, sensitizing tumor cells to anti-HER2 treatments [139]. For ER-positive breast cancer, entinostat reversed endocrine therapy resistance in a xenograft model by reducing HER2 expression [140]. However, in the phase 3 clinical trial, entinostat did not improve the overall survival of ER-positive breast cancer patients resistant to endocrine therapy [141]. Moreover, entinostat in combination with azacitidine, a DNA methyltransferase inhibitor, showed limited benefits to breast cancer patients in a phase 2 clinical trial [142]. Recent early-phase clinical trials are investigating the effects of entinostat in combination with immune checkpoint blockade in advanced breast cancer [143] (Table 3).

Tucidinostat is distinct from other HDAC inhibitors because it inhibits HDAC1-3 (Class I) and HDAC10 (Class II). It is approved by the Chinese and Japanese FDAs but not the United States FDA and is currently in many more clinical trials than all other HDAC inhibitors (Table 3). Tucidinostat was shown to promote autophagy and apoptosis in breast cancer cells in vitro and improve the response to doxorubicin in vivo [144]. In addition, tucidinostat was demonstrated to improve the response of AR-positive TNBC to AR antagonists [145]. Extensive clinical trial results have demonstrated that tucidinostat in combination with endocrine therapy provided therapeutic benefits to patients with advanced ER-positive breast cancer, but adverse events from the treatment were a potential concern [146–149].

Besides those in active clinical trials, panobinostat is another pan HDAC inhibitor approved by the FDA and tested in breast cancer. Preclinical studies indicated that panobinostat induces autophagy in breast cancer cells and inhibits TNBC in vitro and in vivo [150, 151]. Panobinostat was also shown to reduce aromatase expression in ER-positive breast cancer and synergize with endocrine therapy [152]. In the published phase 1 clinical trial of panobinostat in combination with endocrine therapy, a partial response was observed with the highest dose [153]. Other clinical trials of panobinostat in breast cancer were terminated, withdrawn, or completed, but with no published results.

In addition to the ones mentioned, HDAC inhibitors such as mocetinostat and abexinostat have also been tested in the preclinical models of breast cancer. Mocetinostat, an inhibitor of HDAC1-3 and HDAC11, induced the expression of tumor suppressor Fyn-related kinase in basal-like breast cancer and showed antitumor effects in those overexpressing HDAC2 [154, 155]. Our previous study demonstrated that mocetinostat in combination with azacitidine reduced the growth of mesenchymal TNBC in vivo [156]. Abexinostat, a pan HDAC inhibitor, was shown to reduce cancer stem cells in breast cancer with low Xist expression [157]. Our previous findings demonstrated that mocetinostat and abexinostat can reverse EMT in in vitro models of mesenchymal breast cancer [156].

Compared to inhibitors of classic HDACs, SIRT inhibitors have not been as extensively studied in breast cancer. SIRT inhibitors MHY2256, Sirtinol, and Salermide were shown to inhibit the growth of breast cancer cells in vitro and in vivo by increasing p53 acetylation to induce cell death [158, 159]. TM, a SIRT2 inhibitor, induced the degradation of c-Myc and thus inhibited the growth of breast cancer cells and xenograft models [160]. Studies also suggested that sirtuin inhibitors might be able to overcome chemotherapy resistance in breast cancer, but those were not recent studies and were limited to in vitro treatments [91]. A more recent study showed that SIRT5 inhibitors have antitumor activity in breast cancer models [161]. However, to date, SIRT inhibitors have not entered any clinical studies.

In summary, despite the positive results seen in the preclinical setting, most HDAC inhibitors did not show impressive results in late-phase clinical studies for breast cancer. Moreover, a recent study suggested that HDAC inhibitors might promote breast cancer metastasis [162]. This again indicates that the roles of HDACs are complex. To improve their efficacy, especially in the clinic, biomarkers and more in-depth mechanistic studies will be needed to further elucidate the effects of HDAC inhibition. In addition, toxicity and selection of the combination therapy should also be considered and addressed in future studies of HDAC inhibitors.

**Table 3:** HDAC inhibitors in currently active clinical trials for breast cancer. All trial information was obtained from clinicaltrials.gov. PD1: programmed cell death protein 1; CTLA4: cytotoxic T-lymphocyte associated protein 4

Drug name	Phase	Conditions	In combination with	NCT number
Vorinostat	1	Relapsed/refractory and/or metastatic breast cancer	PARP inhibitor olaparib	NCT03742245
	1	Operable HER2- breast cancer	Chemotherapy carboplatin Chemotherapy nab-paclitaxel	NCT00616967
Belinostat	1	Metastatic TNBC	CDK4/6 inhibitor ribociclib	NCT04315233
	1	Metastatic breast cancer	PARP inhibitor talazoparib	NCT04703920
Romidepsin	1/2	Metastatic TNBC BRCA mutation-associated recurrent/metastatic breast cancer	Chemotherapy cisplatin Anti-PD1 nivolumab	NCT02393794
Entinostat	3	Advanced/metastatic ER+/PR+/HER2- breast cancer	Endocrine therapy exemestane	NCT02115282
	1	Advanced/metastatic HER2- breast cancer	Anti-PD1 nivolumab and anti-CTLA4 ipilimumab	NCT02453620
Chidamide (Tucidinostat)	2	Metastatic TNBC	Chemotherapy capecitabine	NCT05390476
			Anti-PD1 zimberelimab	NCT05632848
	1/2	Advanced TNBC	Chemotherapy vincristine	NCT05747313
	2	Advanced ER+/PR+/HER2- breast cancer	Chemotherapy nab-paclitaxel	NCT05633914
			PARP inhibitor fluzoparib	NCT05085626
	2	Early ER/PR-low, HER2- breast cancer	Anti-PD1 Chemotherapy paclitaxel	NCT05749575
	1/2	Metastatic/relapsed ER+/PR+/HER2- breast cancer failed CDK4/6 inhibitor treatment	CDK4/6 inhibitor abemaciclib Endocrine therapy	NCT05464173
	1/2		Chemotherapy eribulin	NCT05335473
	2		Endocrine therapy Chemotherapy capecitabine	NCT05411380
	2	Advanced ER+/PR+/HER2- breast cancer with PIK3CA mutation	mTOR inhibitor everolimus Endocrine therapy	NCT05983107

### Immunosuppressive myeloid cells in breast cancer

The tumor immune microenvironment (TIME) of breast cancer can elicit both antitumor and protumor effects [163]. Immunosuppressive cells in TIME can support tumor progression by promoting tumor growth, facilitating immune escape, contributing to metastasis, and affecting treatment response [164]. Myeloid cells are the most abundant infiltrated immune cells in many cancer types, including breast cancer [165, 166]. Immunosuppressive myeloid cells include polymorphonuclear myeloid-derived suppressor cells (PMN-MDSCs), monocytic MDSCs (mMDSCs), and immunosuppressive subsets of tumor-associated macrophages, monocytes, and dendritic cells (DCs) [167–169].

#### PMN-MDSC

PMN-MDSCs resemble many features of classical neutrophils, and most studies indicate that they originate from granulocytic lineage but are immature and pathologically activated [167, 169]. One study by Mastio et al. suggested that monocytic precursors can also differentiate into PMN-MDSCs [170]. The nomenclature of PMN-MDSCs has been controversial and evolved over time. In humans, PMN-MDSCs are defined as CD11b^+^CD33^+^HLA^−^DR^−/low^CD14^−^CD15^+^(or CD66b^+^), and in mice, they are defined as CD11b^+^Ly6G^+^Ly6C^mid/low^ [171, 172]. There were no markers to distinguish PMN-MDSCs and neutrophils in mice, and therefore PMN-MDSCs can only be defined based on functional studies that assess the immunosuppression activity [171]. Our previous study indicated that TANs of neutrophil-enriched breast cancer suppress T cells and should be considered PMN-MDSCs [173]. Recently, CD84 was identified as an emerging marker to identify MDSCs in breast cancer [174]. Multiple studies showed that tumor-secreted cytokines such as granulocyte and granulocyte-macrophage colony-stimulating factors (G-CSF and GM-CSF) skewed the differentiation of hematopoietic cells towards myelopoiesis in the bone marrow (BM) [175–177]. The overproduction of neutrophils in BM leads to neutrophil accumulation in the blood and spleen [173, 174]. The recent study by Alshetaiwi et al. demonstrated that neutrophils become PMN-MDSCs through an abnormal maturation trajectory in the spleen [174]. In contrast, our previous study showed that BM neutrophils in mammary tumor-bearing mice are immunosuppressive [173]. The study by Patel et al. suggested that BM neutrophils become immunosuppressive only in mice bearing late-stage tumors, providing a possible explanation for different findings in the two studies [178]. How BM neutrophils acquire immunosuppressive activity still needs to be further elucidated. PMN-MDSCs are recruited to the mammary tumor by chemokines such as C-X-C motif chemokine ligand 2 (CXCL2) [179, 180]. In tumors, PMN-MDSCs inhibit antitumor immune cells such as cytotoxic T lymphocytes (CTL) by producing reactive oxygen species and arginase 1 (Arg1) [172]. They also promote the activation and expansion of immunosuppressive regulatory T cells (Tregs) [181]. Breast cancer highly infiltrated with PMN-MDSCs is resistant to immune checkpoint blockade (ICB) [173]. Besides affecting immune cells, PMN-MDSCs also promote breast cancer initiation and support metastatic outgrowth by reverting the EMT phenotype [180, 182–184].

#### Monocytes and mMDSCs

Monocytes can give rise to macrophages and DCs, but some tumor-associated monocytes can be immunosuppressive without differentiation [169]. Monocytes can be categorized into classical and non-classical monocytes. Classical monocytes are defined as CD14^high^CD16^−^ (human) or CD11b^+^Ly6G^−^Ly6C^+^ (mouse), whereas non-classical are defined as CD14^low^CD16^+^ (human) or CD11b^+^Ly6G^−^Ly6C^low^ (mouse) [171, 185]. Classical monocytes exhibiting an inflammatory phenotype were shown to suppress CTLs and facilitate tumor metastasis in breast cancer models [186–188]. Those monocytes are recruited to the tumor by C-C motif ligand (CCL) 2 - C-C motif chemokine receptor 2 (CCR2) signaling [187]. In contrast, non-classical monocytes were demonstrated to inhibit breast cancer metastasis [189].

Tumor-associated mMDSCs are very similar to classical monocytes in marker expression. Human mMDSCs are defined as CD11b^+^CD33^+^HLA-DR^−/low^ CD14^+^CD15^−^ [172]. Mouse mMDSCs were defined as CD11b^+^Ly6G^−^Ly6C^+^, and those classic markers cannot distinguish mMDSCs from monocytes as both originate from monocytic precursors [172]. Similar to PMN-MDSCs, mMDSCs are immature myeloid cells and are the result of tumor-dependent abnormal cell activation. The study by Alshetaiwi et al. indicated that CD84 can also be used to identify mMDSCs from monocytes in breast cancer, in addition to distinguishing PMN-MDSCs from neutrophils [174]. Similar to that of PMN-MDSCs, the first step of mMDSC production is the abnormal expansion of BM myeloid cells driven by tumor-derived cytokines such as G-CSF, TGF-β, and IL-34 [190, 191]. Those cytokines also promote the immunosuppressive activity of mMDSCs. The release of mMDSCs from BM was shown to be regulated by tumor-derived factor PTH1R in a breast cancer model [192]. However, studies demonstrated BM and spleen mMDSCs of mammary tumor-bearing mice are not immunosuppressive, suggesting that they gain immunosuppressive activity when they reach the tumor [192, 193]. In addition, the study by Calvert et al. indicated that tumor mMDSCs have a limited ability to differentiate, while the study by Biswas et al. suggested that exosomes secreted by mesenchymal stem cells can promote differentiation of mMDSCs to protumor TAMs in breast cancer [193, 194]. The recruitment of mMDSCs and monocytes is facilitated by CCL2 and CCL5 in breast cancer [195]. T-cell suppression by mMDSCs is driven by the production of nitric oxide and Arg1 [195]. Moreover, recently, a study by Sarkar et al. showed that mMDSCs suppress CTLs by releasing adenosine in mouse models of multiple cancer types including breast cancer [196]. This study also showed that increased adenosine levels were a result of CD73 expression, which was induced by tumor-derived prostaglandin E2. Furthermore, mMDSCs also induce EMT in tumor cells to support the dissemination and accordingly metastasis in mouse models of breast cancer [184]. Elevated levels of mMDSCs have been correlated with poor clinical outcomes in patients with metastatic breast cancer [197].

#### Tumor-associated macrophages

Tumor-associated macrophages (TAMs) are defined as CD11b^+^Gr-1^−^F4/80^+^ in mice and CD14^+^CD68^+^ in humans. Many early studies categorized TAMs into M1-like (antitumor) and M2-like (protumor) TAMs, but the field has realized that this binary system is oversimplified [198, 199]. Recently, the development of single-cell omics has further revealed the heterogeneity in TAM phenotypes and complexity in TAM biology [200]. In breast cancer, TAMs can arise from both tissue-resident macrophages and monocytes recruited to the tumor by tumor-derived cytokines [201, 202]. High TAM infiltration is associated with the more aggressive Claudin-low subtype of breast cancer, an EMT signature expression, and worse prognosis [173, 185]. The immunosuppressive activity of TAMs in breast cancer was reported in many early studies dating from more than 15 years ago. TAMs inhibit T cell response in breast cancer TIME by downregulating nitric oxide synthase gene expression and upregulating the production of Arg1 and hypoxia-inducible factor (HIF)-1α [203–206]. Recent studies, however, have mostly focused on other tumor-promoting roles of TAMs. A subset of breast cancer TAMs has been found to accumulate in hypoxic regions of mammary tumors and display a proangiogenic phenotype by activating the HIF-2α pathway and VEGF expression [207–210]. Many studies have demonstrated that TAMs induce breast cancer metastasis by promoting cancer cell migration, intravasation, and seeding at the metastasis as reviewed by Williams et al. [201]. In addition, TAMs can induce stem cell-like phenotypes in breast cancer cells through both paracrine and juxtracrine signaling [211, 212]. Because of their roles in tumor progression, inhibitors that deplete macrophages such as colony stimulating factor 1 receptor (CSF1R) antibodies are currently being tested in clinics for cancer treatment [201, 213]. However, besides promoting tumor progression, TAMs have the potential to exhibit tumor-inhibitory phenotypes [200, 213]. Therefore, as reviewed by Rannikko and Hollmen, therapeutics have been developed to reprogram TAMs by targeting various regulatory receptors and metabolic enzymes [214]. HDACs were mentioned as a potential target for TAM reprogramming.

#### Dendritic cells

DCs consist of three different subtypes, plasmacytoid DC (pDCs), conventional DC (cDCs), and monocytic DC (moDCs) [215]. While most DC arise from myeloid progenitors in BM, some pDC can differentiate from lymphoid progenitors [215, 216]. Although pCs can produce interferon and are involved in anti-viral immunity, they have been shown to facilitate immune tolerance in cancer settings [216, 217]. In breast cancer, tumor-derived factors such as tumor necrosis factor-α (TNF-α) reprogram pDCs leading to impaired interferon (IFN)-α production [218, 219]. The reprogrammed pDCs then promote Treg expansion by expressing forkhead box O3 (FOXO3) and inducible costimulatory molecule ligands, and therefore pDCs infiltration is correlated with poor prognosis in breast cancer patients [220–223]. In contrast, cDCs arise from DC progenitors in BM and can be divided into two subtypes cDC1 and cDC2 [215]. Because of their antigen presentation and T cell priming abilities, the infiltration of cDCs, especially cDC1, has been associated with better prognosis in breast cancer patients [224]. However, the normal functions of cDCs can also be impaired by breast cancer and reprogrammed to promote immunosuppression [217]. In the PyMT breast cancer model, cDC1 was shown to highly express the immune inhibitory receptor TIM-3, inhibiting T cell recruitment [225]. Distinct from other DCs, moDCs differentiate from monocytes during inflammation and cancer [215, 217]. In breast cancer, the functions of moDCs have not been widely studied, but one study demonstrated that moDCs from breast cancer patients induced the proliferation of Tregs but not immunostimulatory T cells [226]. Although some DCs can gain immunosuppressive activity, DCs are important in stimulating antitumor immune response. Therefore, various therapies including those targeting epigenetic modulators have been developed and tested to activate DC-dependent immune response or promote DC infiltration [217].

### HATs and HDACs in immunosuppressive myeloid cells

Histone acetylation can regulate the accumulation and phenotypes of myeloid cells in TIME. The study by Sasidharan Nair et al. indicated that the expression of HAT-associated genes increased in PMN-MDSCs while that of HDAC-associated genes decreased [227]. In addition, HDAC2 was shown to facilitate the conversion of monocytes to PMN-MDSCs by reducing the transcription of the retinoblastoma gene [228]. Several studies investigated the effects of HAT inhibitors in MDSCs. The CBP/EP300 BRD inhibitor GNE-781 reprogrammed both mMDSCs and PMN-MDSCs from an immunosuppressive phenotype to a more inflammatory phenotype by inhibiting the expression of STAT-related genes, Arg1, and inducible nitric oxide synthase [229]. Our recent study also showed that CBP/EP300 BRD inhibition can reduce PMN-MDSCs and inhibit abnormal production of granulocytic progenitors in BM [54]. Moreover, the KAT6A (MYST family HAT) was found to acetylate SMAD3 and H3K23 to induce SMAD3 activation resulting in MDSC (Gr-1^+^) recruitment in TNBC [230]. This study also showed that KAT6A inhibitor WM-1119 decreased MDSC recruitment and activated T-cell response when combined with ICB treatment. Many HDAC inhibitors have been demonstrated to reduce MDSC accumulation and/or functions in various cancer types, as reviewed by Adeshakin et al. [231]. For example, the low-dose HDAC inhibitor entinostat in combination with azacytidine inhibited the migration of mMDSCs and reprogrammed the mMDSCs to be more macrophage-like [232]. The HDAC inhibitor vorinostat reduced MDSC infiltration and activated T-cell response in 4T1 tumors by inducing MDSC apoptosis [233]. This study also indicated that the epigenetic therapy combination inhibited the formation of lung metastases by disrupting the premetastatic niches supported by mMDSCs. Another study by Kim et al. found that entinostat in combination with ICB reduced PMN-MDSCs in the breast cancer model 4T1 and suppressed their function [234]. These findings suggest that HDAC or HAT inhibitors can reduce immunosuppression by MDSCs and be combined with ICB to improve T cell activation.

As mentioned previously, targeting the epigenetic modulators is a potential strategy for TAM reprogramming. The class IIA HDAC inhibitor TMP195 was shown to induce phagocytic and immunostimulatory activities of TAMs in a breast cancer mouse model [235]. The low-dose HDAC inhibitor trichostatin-A promoted antitumor phenotypes in TAMs and showed synergistic effects with ICB [236]. HDACs were found to mediate the downregulation of major histocompatibility complex II (MHCII) expression in TAMs, and HDAC inhibitor treatment restored the expression [237]. High expression of HDAC6 was found to promote protumor phenotypes in TAMs, and HDAC6 inhibition improved the response of breast cancer to ICB in part by stimulating an antitumor immune response [238, 239]. Compared to those of HDACs, very few studies investigated HATs in TAMs. One study from Wang et al. showed that EP300 can facilitate the expression of IL-6, a metastasis-promoting cytokine, in TAMs by increasing the acetylation of histone H3 [240].

Compared to TAMs and MDSCs, how HATs or HDACs affect the DC phenotype has not been extensively studied, especially in the cancer setting. HDAC1 was found to be critical for the development of pDCs and cDC2, and HDAC inhibition led to altered differentiation in bone marrow resulting in no pDC production [241, 242]. In tumor-bearing mice, HDAC1 deletion promoted the activation of cDC1 and CTL in TIME [242]. In contrast, HDAC9 deficiency leads to reduced CD8 + DC infiltration and impaired antigen presentation [243]. In addition, inhibition of HDAC6 was shown to inhibit the production of immunosuppressive cytokine IL-10 in both TAMs and DCs [244].

Overall, these studies demonstrated that HDAC and HAT inhibition can reprogram the phenotypes of tumor-infiltrated myeloid cells. However, the effects of HATs have not been as extensively studied compared to those of HDACs. These findings again emphasize the importance of having more specific inhibitors and highlight the importance of examining the effects on TIME while testing those inhibitors for potential cancer treatment. Tumor-associated immune cells such as immunosuppressive myeloid cells might contribute to whether tumors respond to HAT and HDAC inhibitors. Furthermore, HAT or HDAC inhibitors may facilitate T cell activation, and therefore combination with ICB should be considered in the future studies of those inhibitors.

## Conclusion

In summary, although HATs and HDACs have opposite functions in modulating histone acetylation, inhibitors of both have been investigated as potential treatments for breast cancer. The functions of HATs and their inhibitors were mostly studied in hormone receptor-positive breast cancer. Currently, no HAT inhibitors are being tested in clinics for breast cancer specifically, but novel inhibitors such as those targeting CBP/EP300 BRD recently entered early clinical trials for solid tumor treatment. The novel inhibitors have been reported to be effective in inhibiting breast cancer and immunosuppression, but their effects will need to be further elucidated. Compared to HATs, HDACs have been more extensively investigated across different breast cancer subtypes and in tumor-infiltrated myeloid cells. The functions and targets of different HDACs were demonstrated to be complex and context dependent. Many HDAC inhibitors have been developed but have not succeeded in the clinic, especially as single agents. Current clinical trials mostly focus on testing HDAC inhibitors in combination with standard-of-care therapies. The effects of HAT and HDAC inhibitors on breast cancer alone and in combination with standard-of-care therapies should be more carefully investigated in future studies. Biomarkers may be needed to better identify breast cancer patients that might benefit from those inhibitors. The frequency of tumor-associated myeloid cells may potentially serve as biomarkers.

## Data Availability

No datasets were generated or analysed during the current study.

## Abbreviations

ER: Estrogen receptor
PR: Progesterone receptor
HER2: Human epidermal growth factor receptor 2
TNBC: Triple-negative breast cancer
K: lysine
HAT: Histone acetyltransferase
GNAT: Gcn5-related N-acetyltransferase
CBP: CREB-binding protein
EP300: E1A-associated protein p300
SRC: Steroid receptor coactivator
HDAC: Histone deacetylase
BRD: BromodomainPARP: poly (ADP-ribose) polymerase
AR: Androgen receptor
EMT: Epithelial-mesenchymal transition
TGF-β: transforming growth factorβ
EMT-TF: EMT-inducing transcription factor
VEGF: Vascular endothelial growth factor
IL: Interleukin
CDK: Cyclin-dependent kinase
SIRT: Sirtuin
EGFR: Epidermal growth factor receptor
PD1: Programmed cell death protein 1
CTLA4: Cytotoxic T-lymphocyte associated protein 4
TIME: Tumor immune microenvironment
PMN: Polymorphonuclear
MDSCs: Myeloid-derived suppressor cells
mMDSCs: monocytic MDSCs
DCs: Dendritic cells
G-CSF: Granulocyte colony-stimulating factor
GM-CSF: Granulocyte-macrophage colony-stimulating factor
BM: Bone marrow
CXCL2: C-X-C motif chemokine ligand 2
CTL: Cytotoxic T lymphocytes
Arg1: Arginase 1
Tregs: regulatory T cells
ICB: Immune checkpoint blockade
CCL: C-C motif ligand
CCR: C-C motif chemokine receptor
TAMs: Tumor-associated macrophages
HIF: Hypoxia-inducible factor
CSF1R: Colony stimulating factor 1 receptor
pDCs: plasmacytoid DCs
cDCs: conventional DCs
moDCs: monocytic DCs
TNF-α: Tumor necrosis factor-α
IFN: Interferon
FOXO3: Forehead box O3
MHCII: Major histocompatibility complex II
